# Systematic literature reviews in migration studies: approaches to context-sensitive synthesis

**DOI:** 10.1186/s40878-025-00502-7

**Published:** 2025-11-22

**Authors:** Tone Sommerfelt, Mathilde Bålsrud Mjelva, Jørgen Carling, Maryam Aslany

**Affiliations:** 1 Peace Research Institute Oslo (PRIO), P.O. Box 9229, Grønland, Oslo, NO-0134 Norway; 2 The MacMillan Centre for International and Area Studies at Yale, Henry R. Luce Hall 34, Hillhouse Avenue, P.O. Box 208206, New Haven, CT 06520-8206 USA

**Keywords:** Comparative synthesis, Interpretation, Migration processes, Qualitative studies, Survey research

## Abstract

With a burgeoning literature on migration, taking stock of what is known about a topic, pattern, or process is challenging. In many scientific fields, systematic literature reviews (SLRs) are a widespread response to this need. In migration research, SLRs are relatively rare. Most existing literature reviews are non-systematic and often geographically specific. The rarity of comprehensive SLRs might partly reflect migration researchers’ sensitivity to context-specificity and variation, and their well-founded scepticism towards empirical generalizations. In this article, we make a case for the value of SLRs for synthesizing quantitative findings on migration across diverse contexts. However, we foreground the interpretative nature of quantitative research in migration studies and explore approaches to synthesis that recognize exceptions and diversity as much as the search for unifying answers. Our discussion seeks to increase comparability in syntheses across contexts, all the while allowing reviews to convey disparity of findings. In contrast to common advice to narrow down research topics in SLRs, we demonstrate the feasibility of broader review questions. We exemplify our approach with reference to an extensive review of determinants of migration aspirations, drawn from survey-based research, in which determinants were differentiated in terms of the relative consistency and certainty of results.

**Supplementary Information:**

The online version contains supplementary material available at 10.1186/s40878-025-00502-7.

## Introduction

Journals in migration studies now publish well over a thousand articles per year. On top of that come migration-related work in disciplinary, regional, and thematic outlets. This proliferation has produced a rich and diverse knowledge base but also makes it increasingly difficult for researchers and students to take stock of what is known.

A common response to such challenges in other fields is the systematic literature review (SLR), a structured and transparent procedure for identifying and synthesizing existing research. SLRs are widespread and influential in education, medicine, and the social sciences, but remain relatively rare in migration studies. Most reviews in our field are non-systematic and narrative, often confined to specific regions or topics. The notable exception is research at the intersection of migration and health and on service provision among immigrants.

Why are SLRs uncommon in migration research? One reason is that the positivist assumptions of classic SLRs sit uneasily with the interpretative traditions and context-specific processes that dominate the field. If asked, for example, “what does the literature say” about the effects of social networks on immigrant integration, the challenge is not just volume but comparability: how can findings from Colombians in Canada, Lithuanians in Luxembourg and Mozambicans in Malawi be meaningfully synthesized? For socially constituted phenomena, such as those studied in migration research, the pursuit of a single synthesis may even appear futile. Yet we argue that SLRs have considerable potential in migration research, provided they are adapted to our field’s methodological pluralism. Our central question is: how can systematic literature reviews be designed to accommodate the interpretive, context-sensitive nature of migration studies, while still enabling cumulative knowledge building? To address this, we bring debates on systematic reviewing into dialog with the practices of migration research. On the one hand, “rationalist” SLR traditions tend to discount researcher interpretation and overlook contextual conditions. On the other, newer methods for reviewing qualitative and complex materials emphasize narrative synthesis but rarely exploit opportunities for comparative analysis across cases. Migration studies, with its mix of quantitative, qualitative, and conceptual work, offers fertile ground for developing context-sensitive approaches that balance both needs.

We pursue a twofold agenda. First, we demonstrate and discuss methods for synthesising quantitative (survey-based) findings in ways that identify global trends, all the while accommodating diversity of findings and variation across contexts. As part of this discussion, we address the so-called “narrative” syntheses of research results, currently a catch-all category that is usually far less specific in terms of possible procedures for synthesizing statistical results than is meta-analysis. We acknowledge the role of research context in the review process – in the SLR literature a point more often emphasized in qualitative than quantitative materials. Furthermore, we address the analytical inferences of researchers in the review and synthesis process, while simultaneously aiming for transparent forms of synthesis. This involves reframing orthodox SLR methodologies to integrate, rather than suppress, researcher interpretation. This is a point of particular relevance as methods for research synthesis using artificial intelligence (AI) to avoid researcher bias raise new questions of the future role of human-generated SLRs (Block & Kuckertz, 2024).

Second, we enhance comparability and enable reviews to display the comparative consistency and certainty of existing results. We argue that broader, exploratory review questions, though more demanding, can yield comprehensive overviews of scholarly debates, identify knowledge gaps and inspire richer research agendas.

The challenges we highlight are not unique to migration studies, but our field’s methodological inclusiveness makes it a particularly promising setting for developing context-sensitive SLRs. We illustrate this through examples from our own review of research on migration aspirations (Aslany et al., 2021), research summaries carried out in migration studies as well as from other fields. Our goal is not to provide a how-to manual, but to situate migration research within wider debates about systematic reviewing and to propose ways forward for context-sensitive synthesis.

## Systematic literature reviews: approaches and debates

Since the 1990s, scholarship on systematic review methodology has expanded considerably (see e.g. Petticrew and Roberts 2006; Dixon-Woods et al., 2006; Dixon-Woods et al., 2005; Petticrew, 2015; Bradbury-Jones et al., 2021). At its core, an SLR applies transparent procedures for identifying and selecting studies, making inclusion and exclusion criteria explicit. The “systematic” element can also extend to how findings are synthesized, ensuring that the review process is reproducible or at least transparent, depending on the type of research material, review design, and approach.

Early debates in the 1990s reflected sharp contrasts between two traditions. One was rooted in medical and health care research, emphasizing randomized controlled trials (RCTs) and associated with the “evidence-based” rationalist movement of the Cochrane Collaboration (Higgins et al., 2019). The other developed in policy-related and social science fields, where research contexts were more varied and methods more heterogenous. This latter stream included both quantitative and qualitative studies, and highlighted how context, contingency and researcher interpretation shape findings (cf. Dixon-Woods et al., 2006, p. 30).

Over time, methods for synthesizing qualitative research expanded the range of available SLR approaches (see e.g. Bradbury-Jones et al., 2021, p. 2). This diversification sparked ongoing debates about how review questions should be framed. Petticrew and Roberts argued that SLRs can usefully address not only the “what works” question typical of intervention studies, but also broader questions about relationships between variables (2006, 28). While they acknowledged the value of broad reviews for generalizability spanning multiple settings, populations, and formulations of interventions – they cautioned against questions so expansive as to become vague (2006, pp. 29, 75). In later work, Petticrew (2015) criticized the narrow focus of “what works”, suggesting instead that reviewers should ask “what happens” under varying circumstances. This reframing emphasises the diversity of impacts across contexts and the value of integrating insights from qualitative analyses, without losing focus (2015, p. 4).

We build on encouragements both to broaden review questions (Petticrew, 2015) and adopt flexible conceptual frameworks (Gough, Oliver, and Thomas 2012). Our contribution, however, centres specifically on quantitative research, and as we outline below, we argue for extending the scope of reviews also by exploring multiple relationships. In the example we base our discussion on, the aim was to comprehensively review all findings on the formation of migration aspirations (Aslany et al., 2021), including how individual factors (e.g. gender or age) and contextual factors (such as the quality of public services and levels of violence and insecurity) help explain who wants to leave and who wants to stay. The approach was open, limited in scope by the dependent variable (migration aspirations) at the core of the review question (“Which determinants have been found to influence migration aspirations in peer-reviewed journal articles?”). The SLR thus involved an element of mapping of all determinants recognized to affect migration aspirations in the existing survey-based literature. In turn, findings on each individual relationship between the identified determinants (independent variables), and the dependent variable, were subjected to review. Without underestimating the work involved, we discuss the potential benefits of broad review questions and argue that they are a fruitful tool to display what is known, and where the gaps lie.

Artificial intelligence is increasingly integrated into SLRs, reshaping the landscape of review methodologies (see Khalil, Ameen, and Zarnegar 2022 for an overview of tools). AI tools enhance the efficiency of literature searches and facilitate the identification of themes and comparable variables across sources. Nonetheless, the role of the human researcher remains indispensable (Lucio et al., 2025). In research synthesis, the role of AI has particularly been discussed within the rationalist traditions of SLRs, where the ideal of “theory free” reviews is often emphasised. While the advantages of AI in research synthesis are evident, especially within the rationalist approaches, a comprehensive discussion lies beyond the scope of this article. In our approach to SLRs in migration studies, we emphasize the human researcher as a vital resource rather than a limitation. As Block and Kuckertz (2024, p. 1978) observe, “there are always alternative ways of theorizing and problematizing” in the social sciences, and divergent insights within and between author teams can enrich the potential of SLRs, also to inspire new research. Therefore, the human factor in conducting SLRs remains central, potentially in productive interaction with AI tools.

## The catch-all category of “narrative synthesis”

This article promotes the value of systematic reviews of survey-based research in migration studies and argues for a comparative and interpretive approach to research synthesis. This position requires some contextualization in the research synthesis literature.

The synthesis of quantitative data in SLRs has conventionally been grouped into meta-analysis and narrative analysis. Meta-analysis of quantitative data involves the pooling of results, and usually, strict standards in calculations of the mean and variability of effect sizes across studies (Field and Gillett 2010, p. 666). Synthesis forms of meta-analysis vary too, but within the body of literature from the Cochrane Collaboration, which dominates the field, discussions remain closely linked to medical and interventionist contexts (see for example Deeks, Higgins, and Altman 2023).

Narrative analysis, on the other hand, is a catch-all category to denote *all* synthesis forms that are *not* meta-analysis. Narrative analysis is often far less specific in terms of procedures for synthesizing results. Outside of the professionalized “rationalist” traditions of the Cochrane Collaboration’s approach, some reviewers of numerically based research rather arbitrarily characterize their own approach as “meta-analysis”, by virtue of using quantitative measures in synthesis, even though the analysis does not involve the statistical combination of results (for an example, see Neumann and Hermans 2017, p. 7 ff.). In other SLRs within migration research, reviewers contrast “systematic reviews” with “narrative reviews” (see e.g. Ghosh & Orchiston, 2022, p. 5). This brings confusion to a new level, as the narrative label conventionally relates to the synthesis method, whereas the “systematic” element refers to the way in which sources are selected. Our wish is not to police the use of “meta-analysis”, but to point out that syntheses of quantitative data that do not involve the statistical combination of results, is not a standardized exercise. In reviews of research based on qualitative sources, the notion of “meta“ conveys entirely different procedures for review, as seen for instance in meta-ethnography (Blacklock et al., 2014).

We observe that methods for the synthesis of quantitative research – that does *not* involve meta-analysis – continue and *remain* an issue mainly with reference to policy and medical interventions, precisely in the “rationalist”, “what works”-context in which they started out (see for instance Campbell et al., 2020). In this tradition, a common critique of narrative synthesis of quantitative research is that it is “opaque and subject to author interpretation, casting doubt on the trustworthiness of a review’s conclusions” (Campbell et al., 2018, p. 1). The realization of researchers’ interpretative efforts and analytical inferences cannot form a basis for critique of review methods involving survey data: Survey methodologies necessarily involve researcher judgements; they are based on theoretical assumptions; and systematic reviews of survey-based data must reflect these facts.

This motivates our agenda to discuss options for synthesis of research in reviews of quantitatively based data on migration that do not allow for meta-analysis. Moreover, we hope to bring the potential of systematic reviews of quantitative data on more complex research materials back into overall debates on migration studies and methodology. We propose forms of narrative syntheses of research with a view to standardizing comparisons, without reverting to positivist ideals or myths about the objective or “atheoretical” systematic review (Gough, Oliver, and Thomas 2012, p. 68). In contrast, we relate actively to the fact that analytical judgements and interpretation pervade each step of the process involved in SLRs (Dixon-Woods et al., 2005, p. 46). As such, the forms of syntheses of research results that we explore are both summarizing and interpretative (Dixon-Woods et al., 2006).

## Current syntheses of research in migration studies

SLRs remain relatively uncommon in migration research. Where they exist, they predominantly address immigrant populations in the post-migration phase. Typical examples are reviews of migrant health and mental health outcomes, often published in medical or public health journals (Aldridge et al., 2018; Jannesari et al., 2020). SLRs on the effectiveness of care delivery within health and social services (Shaw and Funk 2019) also contribute to this body of work, reflecting the rationalist tradition of SLRs in interventionist “what works” contexts (see Aldridge et al., 2018).

A second strand of reviews explores the migration process itself. However, only a few existing reviews of quantitative studies adopt a systematic approach (notable exceptions include Tjaden, Morgenstern, and Laczko 2018; Pagogna and Sakdapolrak 2021; Ghosh & Orchiston, 2022; Sohst et al., 2020; Neumann and Hermans 2017). Syntheses of research on the migration process, whether systematic or not, often adopt a limited geographical scope, for instance, studies focusing on the Sahel (Neumann and Hermans 2017) or migration to Europe (Sohst et al., 2020). Such geographically focused reviews are valuable in consolidating findings. They also underscore the challenges of cross-contextual comparison. Migration drivers, intentions and practices are shaped by highly diverse conditions, making it difficult to summarize overall trends (cf. Obokata, Veronis, and McLeman 2014, p. 119, 122 ff.; Sohst et al., 2020, p. 14). Some reviews also combine a geographical lens with a narrow thematic focus, for example reviews of illegal migration to Europe (Cummings et al., 2015, a non-systematic review), the effect of aid on migration flows (Marchal, Naiditch, and Simsek 2020, a non-systematic econometric analysis) or the impact of information campaigns (Tjaden, Morgenstern, and Laczko 2018). Other topical syntheses include research on climate change and environmental drivers of migration (Obokata, Veronis, and McLeman 2014). Even when addressing narrowly defined topics, however, such reviews often investigate the relation between phenomena that are differently operationalized and that involve differently formulated variables, thus extending the scope of the reviews.

Our interest in review methods for survey data reflects a broader theoretical shift in migration research, from a concern with physical movements to a wider engagement with people’s thoughts and feelings about migration. Research on cognitive and preparatory processes that precede international migration uses diverse frameworks and perspectives (see e.g. Carling & Schewel, 2018; Koikkalainen & Kyle, 2016; van Naerssen and van der Velde 2015; Willekens, 2017). These approaches foreground the distinction between migration “aspirations” and migration “ability” (Carling, 2002) or “capability” (de Haas, 2021). The result has been an expanding body of survey-based studies on people’s desires, intentions and plans to migrate. Yet, existing SLRs on the migration process rarely reflect this shift or make systematic use of the analytical potential embedded in such survey research. For example, in their SLR on environmental migration, Obokata, Veronis, and McLeman (2014) observed that aims to capture motivations in quantitative studies – at the time the reviewed works were carried out – mostly appeared in research using mixed methods, which used “surveys and simple statistical analyses” (2014, p. 128). The authors thus overlooked research based on larger scale surveys on perceptions, aspirations, etc., though the conclusion on a general lack of survey data on perceptions of environmental factors and stress obviously holds true (Aslany et al., 2021, p. 50).

Overall, most existing reviews synthesize research on migration behaviour through studies of actual movements, while aspirations remain underexplored or inferred indirectly from observed migration outcomes (thus neglecting constraints on migration ability) (see e.g. Neumann and Hermans 2017, p. 2). A notable exception is the review by Hagen-Zanker, Hennessey, and Mazzilli (2023) of subjective and intangible factors in migrant decision-making, which draws on both qualitative and quantitative sources. Reviews by Czaika and Reinprecht (2020) and Kuhnt (2019) also address migration drivers across quantitative and qualitative research, though without employing systematic literature-identification procedures. These examples, along with systematic reviews of qualitative and ethnographic material (see e.g. Blacklock et al., 2014), illustrate the potential of reviews to advance broader theorization of migration decision-making and processes.

Alongside systematic reviews that seek to synthesize findings, there are papers that rather set out to systematically map a research field. This is what Pisarevskaya et al. (2019) sought to do for migration studies as a whole. More recently, the systematic and reflexive review by Klöpf et al. (2025) similarly mapped research on migration in West Africa. Mapping a research field means systematically describing patterns such as which topics are being addressed, by whom, with what methods, and in which locations. Such mappings are highly valuable for understanding the foundations of knowledge in a field, and the way in which prevailing insights might reflect the distribution of research efforts. However, they largely evade the challenge we address in this article: how to synthesize substantive findings about migration.

In the following section, we discuss how systematic reviews might be adapted to tackle complex research questions in migration research, focusing in particular on the synthesis of survey-based material. Following insights from the literature on reviews of qualitative research, we reject claims of “theory free” SLRs (cf. Gough, Oliver, and Thomas 2012, p. 68). Instead, we argue that reviews of survey data require a non-positivist approach that acknowledges the interpretive character of both research and research synthesis (Petticrew and Roberts 2006, p. 76). While we do not detail procedures for literature selection (described thoroughly inMoher et al., 2009; Page et al. 2021), we emphasize that inclusion and exclusion criteria must be transparent, as they shape the biases embedded in the final corpus of literature (Wanyama, McQuaid, and Kittler 2021). Finally, the shift towards “migration aspirations” raises terminological challenges. Terms such as migration aspiration and intention, potential migration and willingness to migrate are used interchangeably across disciplines (Carling & Schewel, 2018; Carling, 2019). Since migration studies is highly interdisciplinary, with economists, psychologists, and sociologists also publishing on related topics in non-migration journals, SLRs must account for such terminological diversity when designing search strategies, ensuring that synonym sets are sufficiently broad to capture relevant work.

## Exploring variables: identifying comparable results

Building on the encouragements in the SLR literature discussed above, we argue for extending the “broadness” of reviews in three ways:

1. From outcomes to processes, moving from “what works” to “what happens” across research contexts.
2. From narrow to umbrella like-variables, considering broad categories of determinants.
3. From single to multiple relationships, examining how several independent variables affect a given dependent variable.

The third dimension is particularly uncommon in SLRs and radically extends the scope of reviews.

As migration research typically deals with broad analytical concepts, a closer examination of how variables have been operationalized is a necessary step to identify the basis for comparison of results. Consider labour market integration, political participation, and transnational ties, for instance. Each concept can be roughly differentiated from strong to weak but could, justifiably, also be divided into qualitatively different categories. Such choices in an SLR depend on analytical reasoning and the diversity of the literature at hand, which influence conclusions. If studies of, say, transnational ties have been conducted in ways too diverse for comparison, one option would be to analyse the effects on social and economic ties separately. Even when the concept for measurement appears self-evidently coherent, the formulation of questions – also in local vernaculars – shapes responses. Moreover, choices of terms for comparison, and operationalizations, are inherently analytical.

In broad and explorative SLRs, another challenge involves clustering differently operationalized determinants into higher-order thematic domains (Czaika and Reinprecht 2020). We suggest a bottom-up process of identifying related variables with a top-down process of drawing inspiration from analytical frameworks. In our SLR, we settled on five overarching thematic domains: Demographic and family related factors (seven determinants), socio-economic factors (six), other individual level factors (seven), conditions related to the country or community of origin (eight), and migration-related factors (four). We also added a residual domain of thematically unrelated variables that did not fit under any of the other themes. See Table 1 in the online Supplementary Information for an overview of the overarching thematic domains.

The clustering into thematic domains is the starting point for the process of deciding which variables should be merged for systematic review. Some measures are relatively unambiguous, such as *age* or *homeownership* (although notions of ‘ownership’ obviously vary). In other cases, thematically related measures differ profoundly, and merging becomes an acute analytical issue. This applies to *employment status* and *violence and insecurity*, where determinants cannot be established without merging diverse measures and operationalizations (cf. Aslany et al., 2021). However, if merged constructs are too broad and encompass radically different measures, they lose their analytical value. If they are too narrow, the number of data points for each determinant might be too small, and in effect, variables in the review run the risk of becoming unwieldy. This balancing act can be eased by including residual categories for measures that defy merging, such as *social identities* and *personality traits* in our case. As we show later, synthesis form can be adapted accordingly.

The thematic domains that we identified were broken down into 33 independent variables, with a minimum of three and a maximum of 70 analyses covering each one (for a detailed overview, see Table 1 of the online Supplementary Information). Our subsequent review involved the synthesis of research on each of the 33 variables’ effects on migration aspirations. In migration studies, research is typically defined in geographical terms, and a single study might report results from several geographical units or national groups side by side. Consequently, the number of *analyses* to be included in the review might be larger than the number of publications, or studies. For instance, a single study might report separately about Angolan, Brazilian, and Romanian immigrants in Portugal, and thereby yield three distinct units of analysis in the SLR.

Beyond the specification of each variable, there are two other potential limits to comparison: *differences between the samples* and the inclusion and omission of *other independent variables*. Firstly, the criteria for sample selection can subtly change the meaning of apparently identical variables. Age is a case in point. In a sample of 18–39-year-olds, we might expect age to have a positive effect on migration aspirations. But even in the same population, a sample of 18–75-year-olds might show no effect of age. This is because the actual effect of age across the adult life span is often non-linear. Comparing results from the two samples would misleadingly give the impression of divergent results.

Secondly, in multivariate analyses, the meaning of each variable depends on all others. A good illustration from migration studies is the so-called remittance decay hypothesis, which posits that remittance flows decline over time. Imagine comparing analyses of survey data on migrants that have remittance-sending as the outcome and years since migration as an independent variable. ‘Years since migration’ is a relatively straight-forward variable on its own, but its meaning changes drastically if variables on transnational kinship ties are also included. In short, migrants often keep sending remittances for many decades as long as there is someone to send to, and stop when the recipients pass away or migrate (Carling, 2022). Controlling for these factors, years since migration might become insignificant. Again, two analyses with different specifications might therefore appear to produce different effects of the same variable, even when the processes at work are the same.

More generally, the social phenomena that migration research explores are not naturally given, and they cannot be strictly delineated or standardized. Consequently, regression results can only be combined and synthesized in meta-analysis under very strict conditions. This applies especially to analyses based on survey data. The variation in terminologies and operationalizations of key terms makes the review process of survey data subject to analytical decisions, and in broad reviews, these analytical decisions are multiplied. The harmonization of different formulations of variables into comparable determinants – both with reference to the independent and dependent variables – illustrates the inevitable interpretative character of reviews that aim to summarize or “integrate” results (cf. Dixon-Woods et al., 2006).

## **Synthesizing results**: visualization and narration

The aim of the synthesis of findings in an SLR in migration studies is not a simple global trend analysis: Rather, syntheses should convey diversity of data material and coverage, as well as diversity of findings in terms of effects and significance levels. Research contexts inevitably affect the potential interpretation of findings and demonstrate the problems of calculating cross-study summary effects. A typical example relates to the impact of household size on migration aspirations. If the impact of household size on persons’ inclination to want to migrate varies systematically in different contexts, it may, for instance, indicate differences in household labour needs in different economies. Calculating cross-context summary effects in this situation does not necessarily contribute meaningfully to a synthesis of insights of the mechanisms that influence human migration.

We propose three different types of synthesis, two of which involve standardized comparisons and visualization of results. Data visualization is increasingly valued in research communication, and communication of data insights more broadly, also in the field of migration (Abel and Sander 2014; Allen, 2023). However, visualizations have been criticized for their purported objectivity and suppression of critical perspectives (Hill, Kennedy, and Gerrard 2016). In the context of an SLR, we argue that visualization can provide a rich form of synthesis that presents an overall pattern – or the lack of it – while also drawing attention to exceptions, outliers, and sub-patterns such as differences between geographical regions. Moreover, visualizations can effectively convey the solidity of the foundations for drawing conclusions and is a synthesis form that is particularly well suited to SLRs that seek to acknowledge disparities in findings and their certainty.

A major limitation in visualizing SLR results is the amount of *comparable* information that is available from each analysis. It might be as little as the direction of the effect, some measure of significance, and the sample size of the survey. However, even this limited information can, as we will show, be meaningfully visualized.

Based on the number of analyses and the level of comparability of the results, we suggest synthesizing results for each determinant in one of three ways:

- *Comparison and visualization of effect sizes and significance levels*: Relatively unequivocal binary determinants allow direct comparisons of effect sizes and significance levels across analyses.
- *Comparison and visualization of significant effects by direction*: Whether variables have positive or negative impacts on the outcome, can be compared and visualized, even if the sizes of effects are not comparable.
- *Stand-alone narrative synthesis and comparison*: When the analyses of determinants are too few or the measurements too disparate to be summarized numerically and visually, synthesis can be made through narration.

Below, we elaborate on each form in some detail.

### Comparison and visualization of effect sizes and significance levels

The direct comparison of effect sizes should be done with caution. Effect sizes cannot be compared directly in circumstances when:

- The analyses use different regression methods. Effects from, for instance, logistic regression are on a different scale than from Ordinary Least Squares (OLS) regression and should thus not be compared directly against each other.
- The operationalizations of the variables are too disparate.
- The response categories are dissimilar. As an example, many of the analyses reviewed in (Aslany et al., 2021) use a variable on educational attainment, but as educational systems vary from country to country (and sometimes within countries), we could not compare the effect of going from one educational level to the next directly but compared the directions of effects – whether more education impacted migration aspirations similarly across the analyses.

Operationalizations of some determinants in existing studies enable direct comparison of effect sizes, for instance related to gender and parenthood. In our own SLR, most of the analyses that investigated these determinants used binary measures (male–female; having children–not having children). This enables direct comparison of effect sizes and significance levels. However, binary measures of parenthood, for example, are not directly comparable to numeric or categorical measures (e.g. indicating the respondent’s number of children). If the data allows, one may include multiple effect size comparisons, use different modes of syntheses for different operationalizations, or classify them under different higher-order themes. These choices, again, highlight the interpretative nature of the mapping and identification of higher-order themes.

**Fig. 1:**
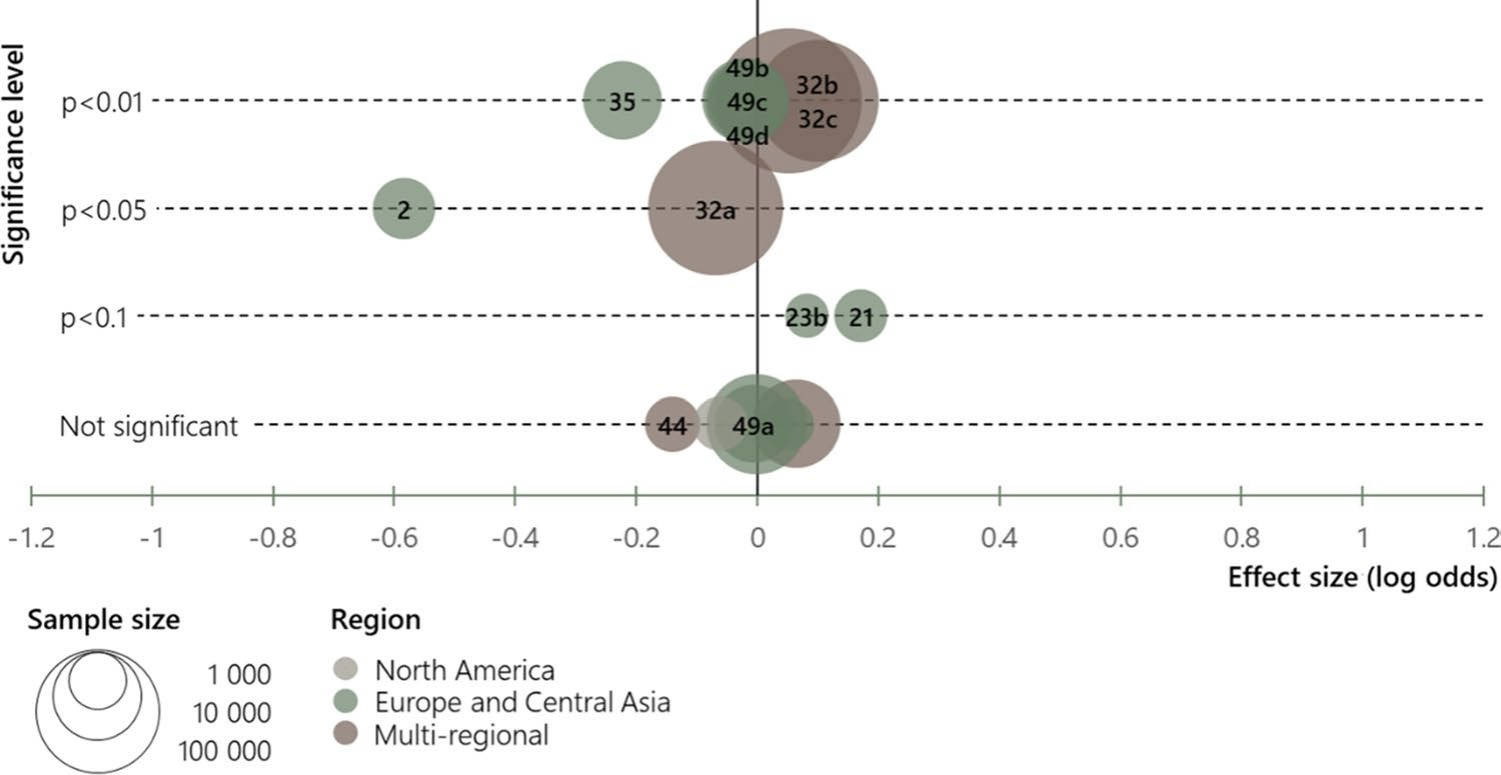
Effects of parenthood on migration aspirations. Each number refers to a specific analysis included in the SLR (Aslany et al., 2021, p. 12–14). Analyses may differ as to whether or not effects with *p* < 0.1 are identified as such or reported as not significant. In some cases, log odds displayed here are converted from odds ratios in the original publication and/or inversed for consistent measurement across all analyses. Articles 21, 23, and 35 use probit models which limits their comparability with the other effects. The figure is produced using MS Excel

Figure 1 shows the visual presentation of the effect of being a parent compared to not being a parent on migration aspirations. Effect sizes are shown along the horizontal axis, and significance levels on the vertical axis. Each bubble represents one analysis, identified through numbers (Aslany et al., 2021, pp. 12–14). The placement of each bubble thus conveys the effect size and the significance level. Since most sources report only significance levels and not exact probability values (p-values), the vertical distribution of the bubbles is not continuous. For the same reason, the figure does not display confidence intervals. The size of the bubbles signifies sample size, which is important for interpreting the overall pattern of results. The colours of the bubbles show the geographic focus of the analyses.

In the case of parenthood, analyses that did not use one or more binary measures were excluded from the figure, but we elaborated on their results in a general discussion of the literature on parenthood and migration aspirations.

Figure 1 conveys diversity of findings on global patterns by highlighting the geographical dimension and studies’ sample sizes. It does not convey whether the studies were based on a national, subnational, or multinational sample, but gives an indication of the geographic distribution of the literature studying this determinant. Figure 1 does not convey an overall or clear pattern in the effect of parenthood on migration aspirations, implying that the role of parenthood is context dependent.

There are, of course, other alternatives to visualise effect sizes. The most common in the context of SLRs are so-called forest plots, which display all effect sizes and confidence intervals for bivariate relations on a comparative scale (see e.g. Aldridge et al., 2018). Forest plots are only feasible when effect sizes are directly comparable, and confidence intervals (or the underlying statistics) are reported. Conventionally, plots do not convey sample size, though occasionally display the weight given to each study, which could be based on the size of the sample (Borenstein et al., 2009). Other metadata, such as geographical scope, could potentially be included in the forest plot, but this is uncommon.

Forest plots are typically used in conjunction with the calculation of a summary effect across the reviewed studies in a meta-analysis (Borenstein et al., 2009). Calculating a summary effect could have been possible in our review for the variables where we directly compare effect sizes. However, it is problematic to quantify a summary effect across diverse contexts that do not make up a meaningful population. For instance, it may be interesting to compare three studies from Romania, Peru, and Latin America overall, but misleading to compute a summary effect.

### Comparison and visualization of significant effects by direction

Many studies in migration research use identical or similar variables, but operationalizations or model specifications differ in ways that prevent direct comparison of effect sizes. The disciplinary diversity of migration studies contributes to variation in the types of models that are employed and the specific statistics that are reported. For example, even a dozen studies of the effect of migration background on labour market participation could use measures that render direct comparison of effect sizes impossible. The only comparable information often turns out to be the *direction* of the effect, its statistical significance, and the survey’s sample size.

**Fig. 2:**
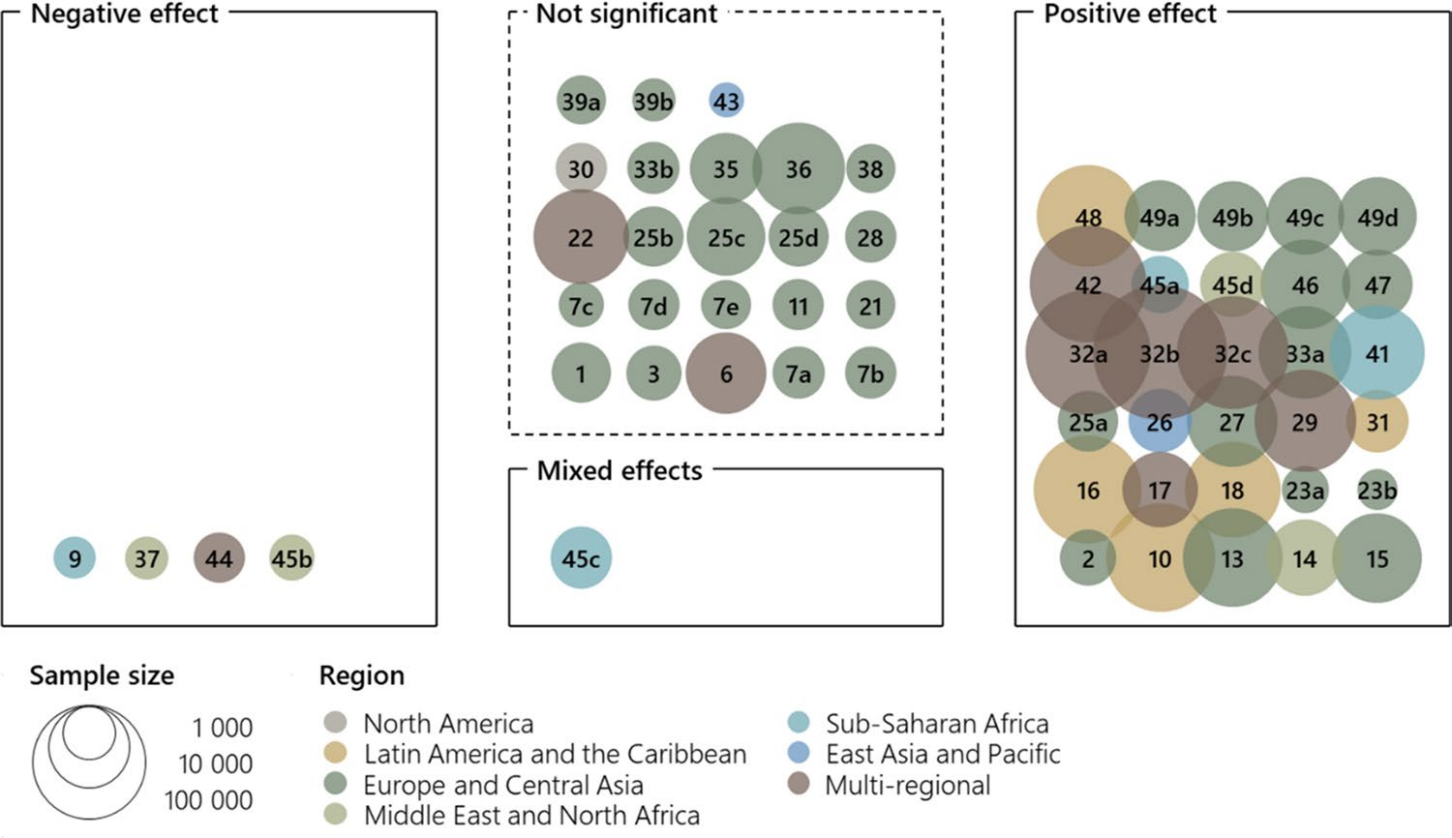
Effect of educational attainment on migration aspirations. Each number refers to a specific analysis included in the SLR

This was the case for most variables in our SLR of determinants of migration aspirations. We chose to visualize the available comparable information, as exemplified in Fig. 2, which shows the effect of educational attainment on migration aspirations. 58 analyses included educational attainment as an independent variable, but they varied in the ways that educational attainment was measured – in binary terms, with categories, or as number of years. Yet, the effects could be divided into four categories: significant positive effects, significant negative effects, significant mixed effects, and non-significant results. Mixed effects include results with significant effects in both directions, e.g., showing a curvilinear relationship. In technical terms, mixed effects appear when the independent variable is categorical or if it is additionally included in squared form. Like Figs. 1 and 2 shows the sample sizes and geographic scope of the survey. The visualization standardizes the comparison of findings that would otherwise be summarized narratively. It enables discussion of overall trends as well as diversity and exceptions.

As apparent in Fig. 2, there were a handful of negative effects of educational attainment on migration aspirations, one case of mixed effects, and many insignificant results. However, most analyses observed a positive impact of education on aspirations to migrate. The visualization also shows that many of the non-significant effects stem from studies focusing on Europe or Central Asia, which suggests potential regional variations in the effect of education.

The synthesis does not show systematic variation by region. It is worth noting, however, that all analyses from Latin America and the Caribbean found positive effects, and that results from Sub-Saharan Africa and the Middle East and North Africa are particularly divergent. Such observations demonstrate that a visualization can be a starting point for inquiries into diversities and their possible explanations. Even so, figures should not be left to speak for themselves but be accompanied by an interpretative narrative.

### Stand-alone narrative synthesis and comparison

In some cases, the effects of a given variable, or a cluster of related ones, do not lend themselves to standardized comparative synthesis. This applies in three situations. First, variables might be measuring different phenomena, even though they appear as similar. In our review, *personality traits* is a case in point. We merged relevant results under this heading, but they diverged in terms of which traits had been considered, and how they had been measured. A second, and often overlapping, issue is that many variables lack a clear directionality, such as religious identity. Third, the number of studies that include a given variable might simply be too small to warrant systematic comparison through visualization. We chose to visualize results when there were at least five observations.

We argue that in a broad and explorative SLR, it is essential to give proper consideration also to determinants that do not lend themselves to standardized comparison. Uncommon variables might represent innovation and be particularly important to examine in future studies. As an example, within the realm of personality traits, one study has found that *self-efficacy* increases migration aspirations, whereas another, perhaps surprisingly, found *perseverance* to have a negative effect.

When standardized comparison and visualization are not feasible, synthesis must take the form of a stand-alone narrative. We refer to it as “stand-alone” because it is unaccompanied by a comparative visualization. Such a synthesis can involve both summary and comparative discussion of findings. For instance, our review included 12 studies with a measure of social identities among the determinants of migration aspirations, spanning ethnicity, religious affiliation, and racialized identities. It would not be possible to systematically compare, for instance, the effect of Asian versus European-origin ethnic identity in Kyrgyzstan (Agadjanian, Nedoluzhko, and Kumskov 2008), Russian versus Latvian ethno-linguistic background in Latvia (Ivlevs, 2013), Sunni versus Shi’ite Muslim or Christian identities in Iraq (Ozaltin et al., 2020), with the relative importance of religion in people’s lives in Sub-Saharan Africa (Sadiddin et al., 2019). Yet, a narrative summary of diverse findings in different contexts can provide insights and identify gaps in the literature.

## **Comparative synthesis**: relative consistency and certainty

So far, we have discussed syntheses of bivariate relationships between one independent variable and the outcome of interest. As mentioned, most SLRs address one such relationship in depth. In contrast, broad reviews, which consider a wide range of independent variables, implicitly raise the question of which factors have the most consistent impact on the dependent variable, across studies and contexts.

In the absence of comparable information on effect sizes, how can this question be answered? We propose to focus on variation in the *consistency* and *certainty* of analysis results. Both are assessable for variables that lend themselves to standardized comparison and visualization.

Visualizations of the kinds exemplified in Figs. 1 and 2 show whether effects are mostly positive, mostly negative, or divergent. To facilitate comparison of effects of different variables, we suggest inverting the variables that lean towards negative results, so that all can be assessed in terms of the strength of effects in one direction. Thus, if “satisfaction with government” has mostly negative effects on the outcome, it should be inverted into “dissatisfaction with government,” which has a mostly positive effect.

Once the variables are aligned in this manner, the degree of consistency in positive effects on the dependent variables can be assessed. We deliberately opt for an holistic and interpretative approach rather than a mechanical or mathematical procedure in this assessment. Based on an analytical reading of the individual figures of bivariate relationships in our SLR, we applied a five-fold classification: divergent, slightly positive, mainly positive, overwhelmingly positive, and consistently positive effects. We also allowed for differentiation within each category. This assessment should take all the available information into account. Thus, the determinants that “overwhelmingly” point in one direction can be distinguished by the nature of the exceptions to the overall trend. If there is one single exception, it matters whether it is represented by a very large multi-country survey or a survey that covers a few hundred respondents in a single city. If there are several exceptions, they will weigh more heavily if they represent different studies and different geographical regions.

Using the example of educational attainment (Fig. 2), the effect on migration aspirations does not point consistently in one direction. A large share of analyses has found a positive effect, but there are many insignificant results and five analyses that have found negative or mixed effects. However, these divergent results were all based on small samples. On our five-point scale, educational attainment was ultimately classified as having a “mainly positive” effect on migration aspirations. That is, it was stronger than “slight” but not as strong as “overwhelming”, given the many insignificant and divergent results. An essential quality assurance mechanism in this process is to review all the figures that have been given the same classification and assert that they indeed represent a similar level of consistency.

This approach differs from the mainstream tradition of SLRs, which, in a positivist fashion, seeks to minimize “subjective” judgements. We argue that the best measure of consistency in a field like migration studies can be generated by combining the rule-based, systematic comparison of empirical results with the analytical assessment of researchers. However, the parameters of this assessment should be made explicit and as transparent as possible.

In meta-analyses, which presuppose directly comparable results, the scope for mathematical summary is greater. Studies can be weighted by factors such as their geographical scope and sample size (Borenstein et al., 2009, p. 5). This approach does not accommodate analytical judgements of the nature and importance of the exceptions. An automated weighing of results can give an impression of straightforward objectivity and under-communicates the interpretive dimensions of review efforts.

The second dimension we assess is *certainty*, referring to the volume of empirical results that underlie the syntheses. We adopt a simple, mathematical approach, starting with the number of analyses that report on each variable. As noted earlier, the number of analyses is greater than the number of articles for review, since some articles report separate analyses on different samples, e.g. separated by country. In the measure of certainty, we assign a lower weight to analyses reported in the same article. This is because *independent* results represent a broader and more certain foundation than results that are based on the same variables and model specifications, even if they use different samples. There is potential for more elaborate measures of certainty, but the overarching variation in our case is the span from five observations for internet use and remittances, for instance, to more than ten times as many for age, gender and educational attainment.

**Fig. 3:**
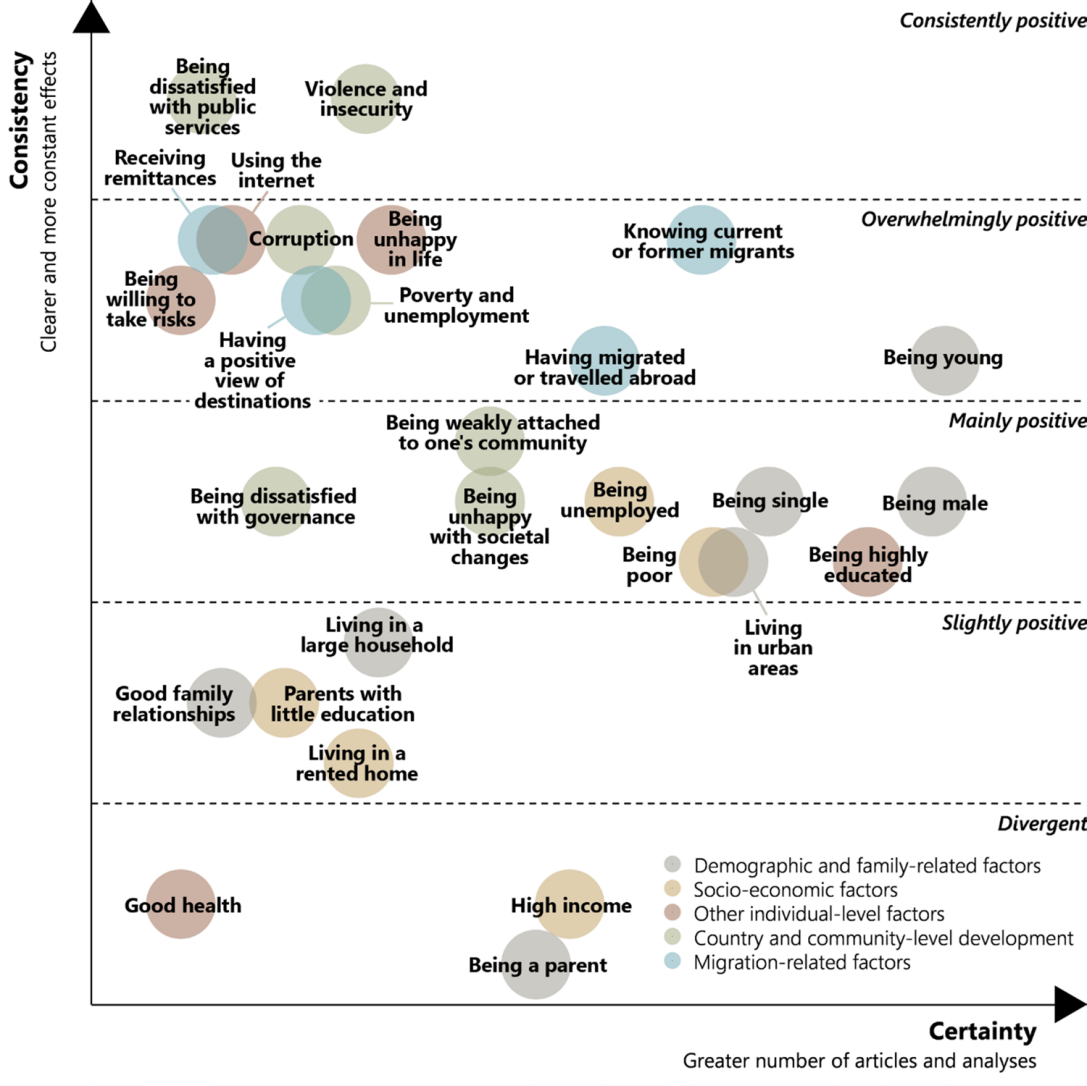
Comparative overview of the effects of determinants of migration aspirations. Taken from Aslany et al. (2021)

When consistency and certainty are established, the variables can be compared as in Fig. 3, which to our knowledge is a new way of visualizing results. Each variable is formulated in a simplified way that clearly conveys the direction of measurement. The top-right corner of the figure gathers the most certain and consistent effects. Being young (i.e. the inverse of higher age) and knowing current or former migrants are approaching this corner of the figure. In the top-left corner we find effects that appear to have consistent effects but based on quite narrow empirical foundations. These might be variables that merit particular attention in future studies.

Importantly, the figure does not show which determinants matter the most, in the sense of having the strongest effect on migration aspirations. However, it shows where the literature is unison regarding the direction of the effects, where it is not, and where more research is needed. Just like the visualizations of bivariate relationships, this figure does not present results exhaustively but provide a foundation for discussion and interpretation.

## Conclusion

As the body of knowledge in migration studies grows, so does the need for appropriate ways of taking stock of insights from existing research. SLRs can play a greater role in the response to this need. Guidance on SLRs from other fields is valuable but is often geared towards other types of data and has positivist and generalizing ambitions that are at odds with mainstream migration research. This article thus aims to fill a gap by demonstrating ways of doing SLRs that respect the interpretive dimensions of our knowledge and the context-specificity of migration processes.

The aim of SLRs in migration studies should, in our view, be to (1) provide an overview of the research field – e.g. which factors are included and omitted from consideration, (2) identify overall empirical trends or patterns where they exist, and (3) reflect on diversity, exceptions, and their possible explanations. In this way, the unifying ambitions of classic SLRs are balanced against the reflexive and context-sensitive traditions of migration studies. Irrespectively of modes of synthesis, the distribution of results can be contextualized and interpreted by drawing on historical, ethnographic, and qualitative material. Such contextualization facilitates reflection on the processes that underlie the emergent patterns, as well as on the possible reasons why results differ geographically.

The prospect of being included in an SLR is an additional argument for migration researchers who do quantitative analyses to fully document their methods and results. Regardless of whether data can be shared, background information on, say, sampling criteria, question formulations, and model specifications should be available. A recent overview (Mjelva & Carling, 2023) found that survey data analyses in migration studies are often lacking in this respect. It might not be feasible to report all the relevant details in a journal article, but online supplements or documents in external repositories make it possible.

Another potential for improvement is to reduce the reliance on arbitrary thresholds for statistical significance and, for instance, report p-values rather than significance levels or confidence intervals. This would be in line with the ongoing shift towards what Wasserstein et al. (2019) call “a world beyond ‘*p* < 0.05’” where statistical uncertainty is treated more continuously and analytically. The approach to synthesis that we have shared in this article is, in many ways, stuck in the old world of significance thresholds, as this is how most analyses report their findings. In other words, the fourfold distinction between positive, negative, non-significant and mixed effects is convenient, but not ideal. As authors start reporting uncertainty in more nuanced ways, future SLRs should follow and develop new ways of synthesizing degrees of certainty across analyses.

Most importantly, we encourage migration researchers to continue and further develop exchanges across methodological specializations. SLRs that are based on quantitative research can be strengthened by insights from qualitative studies. Such insights can be used to connect numerical patterns to the real-world complexity of migration processes. The resulting sensitivity to context-specificity, interpretation, and uncertainty should in turn make SLRs more appealing as foundational knowledge also for qualitatively oriented migration scholars. And as scholars adopt new approaches to literature reviewing, we emphasize the value of human interpretation in systematic review processes, and encourage scholars to let AI models complement, but not overtake, the human role in analyses.

## Supplementary Information

Below is the link to the electronic supplementary material.

Supplementary Material 1

## Data Availability

All data generated or analysed during this study are included in this published article and its supplementary information file.
