# Supplementary material for: Systematic literature reviews in migration studies: approaches to context-sensitive synthesis
Source: Comp Migr Stud. 2025 Nov 22;13(1):92. doi: 10.1186/s40878-025-00502-7 (PMC12748309; doi:10.1186/s40878-025-00502-7)
Supplement: Supplementary file 1 — Supplementary Material 1 [file 40878_2025_502_MOESM1_ESM.docx]

Supplementary Information for “Systematic literature reviews in migration studies: approaches to context-sensitive synthesis”

# Overview of thematic domains covered in literature review

Table 1 gives an overview of the overarching thematic domains and related determinants that were identified in the systematic literature review in (Authors 2021). The table also shows the type of synthesis we used to present results on each determinant. For more information on the results from the systematic literature review, see (Authors 2021).

Table 1 Thematic domains and determinants covered in our systematic literature review, with number of analyses and synthesis form

| **Domain and determinant** | **Number of analyses** | **Synthesis form** |
| --- | --- | --- |
| Demographic and family-related factors |  |  |
| Age | 70 | Visualization of direction |
| Gender | 69 | Visualization of effect size |
| Marital status | 43 | Visualization of effect size |
| Parenthood and children | 21 | Visualization of effect size |
| Household size | 12 | Visualization of direction |
| Family relations | 4 | Stand-alone narrative |
| Urban or rural residence | 42 | Visualization of direction |
| Socio-economic factors |  |  |
| Socio-economic status | 35 | Visualization of direction |
| Homeownership | 12 | Visualization of direction |
| Employment status | 27 | Visualization of effect size |
| Income | 23 | Visualization of direction |
| Other aspects of employment or activity | 36 | Stand-alone narrative |
| Parental education | 8 | Stand-alone narrative |
| Other individual-level factors |  |  |
| Educational attainment | 58 | Visualization of direction |
| Social identities | 12 | Stand-alone narrative |
| Health status | 3 | Stand-alone narrative |
| Subjective well-being | 11 | Visualization of direction |
| Willingness to take risks | 3 | Stand-alone narrative |
| Personality traits | 4 | Stand-alone narrative |
| Internet access and use | 5 | Visualization of direction |
| Country and community of origin |  |  |
| Country- or community-level development | 7 | Visualization of direction |
| Governance | 7 | Visualization of direction |
| Public services | 5 | Visualization of direction |
| Corruption | 7 | Visualization of direction |
| Violence and insecurity | 10 | Visualization of direction |
| Social attachment and participation | 16 | Visualization of direction |
| Norms and values | 8 | Stand-alone narrative |
| Change over time | 19 | Visualization of direction |
| Migration-related factors |  |  |
| Migration history | 26 | Visualization of direction |
| Migration networks | 34 | Visualization of direction |
| Remittances | 5 | Visualization of effect size |
| Perception of destinations | 9 | Visualization of direction |
| Other determinants |  |  |
| Other determinants | 19 | Stand-alone narrative |

The table is an elaboration of information given in (Authors 2021)

# References

Authors. 2021. *[Systematic literature review]*.
